# The role of fluid pressure in induced vs. triggered seismicity: insights from rock deformation experiments on carbonates

**DOI:** 10.1038/srep24852

**Published:** 2016-04-26

**Authors:** Marco M. Scuderi, Cristiano Collettini

**Affiliations:** 1 Dipartimento di Scienze della Terra, Sapienza Università di Roma, Piaz. Aldo Moro 5, 00185 Rome, Italy; 2 Istituto Nazionale di Geofisica e Vulcanologia, INGV, via di Vigna Murata 605, 00143 Rome, Italy

## Abstract

Fluid overpressure is one of the primary mechanisms for tectonic fault slip, because fluids lubricate the fault and fluid pressure reduces the effective normal stress that holds the fault in place. However, current models of earthquake nucleation, based on rate- and state- friction laws, imply that stable sliding is favoured by the increase of pore fluid pressure. Despite this controversy, currently, there are only a few studies on the role of fluid pressure under controlled, laboratory conditions. Here, we use laboratory experiments, to show that the rate- and state- friction parameters do change with increasing fluid pressure. We tested carbonate gouges from sub hydrostatic to near lithostatic fluid pressure conditions, and show that the friction rate parameter *(a − b)* evolves from velocity strengthening to velocity neutral behaviour. Furthermore, the critical slip distance, *D*_*c*_, decreases from about 90 to 10 μm. Our data suggest that fluid overpressure plays an important role in controlling the mode of fault slip. Since fault rheology and fault stability parameters change with fluid pressure, we suggest that a comprehensive characterization of these parameters is fundamental for better assessing the role of fluid pressure in natural and human induced earthquakes.

Fluid overpressure is considered one of the primary mechanisms that facilitate fault slip. In the seminal paper by Hubbert and Rubey (1959)^1^, it is proposed that fluid pressure, P_f_, reduces the effective normal stress, (σ_n_ − P_f_), that clamps the fault in place, facilitating fault slip. Building on Hubbert and Rubey’s work, numerous scientific contributions have emphasized the role of fluid pressure in fault reactivation and earthquake triggering^2^,^3^,^4^,^5^. However, the analysis proposed by Hubbert and Rubey discusses the role of fluid pressure in fault reactivation^6^ but it does not address the question of slip behaviour, seismic or aseismic, upon fault reactivation. Elastic dislocation theory combined with rate-and-state friction constitutive equations provide a more comprehensive analysis of fault stability^7^,^8^,^9^,^10^,^11^,^12^. In a velocity weakening fault gouge, frictional instability occurs if the elastic stiffness of the loading system, *k*, is smaller than a critical fault rheologic stiffness, *k*_*c*_, defined by the effective normal stress and the frictional constitutive properties of the fault:


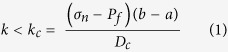


where *D*_*c*_ is the critical slip distance and *(b − a)* is the friction rate parameter. Equation 1 predicts that an increase in fluid pressure reduces k_c_, favoring stable sliding rather than earthquake slip^9^,^11^.

The role of fluid pressure, in facilitating fault reactivation or promoting stable sliding, in frictional stability analysis creates an apparent contradiction in the mechanics of earthquakes. From one side, fluid-assisted fault reactivation and earthquake triggering has been supported by the positive correlation between modelled high-pressure fronts and earthquake locations^5^,^13^,^14^. On the other hand, building on frictional stability analysis^9^,^11^, several works have proposed that pressurized fault portions, imaged as high Vp/Vs domains, are characterized by aseismic slip^15^,^16^. This apparent contradiction of the role of fluid pressure in fault stability poses a serious problem in our understanding of earthquake physics with numerous implications, including a better assessment of the risk of human induced earthquakes^17^. Despite this contradiction, until recently, there have been only a few experimental studies on the role of fluid pressure in fault frictional stability^18^,^19^. However, these studies do not systematically analyse the potential role of fluid pressure in the evolution of rate- and state- friction parameters.

In this work we develop a systematic study to characterize the evolution of friction constitutive parameters *(a − b)* and the critical slip distance, *D*_*c*_, under different levels of fluid pressure, spanning from sub-hydrostatic to near lithostatic fluid pressure conditions. Then we discuss the implications of our findings in the context of fault frictional stability.

## Results

**Figure 1:**
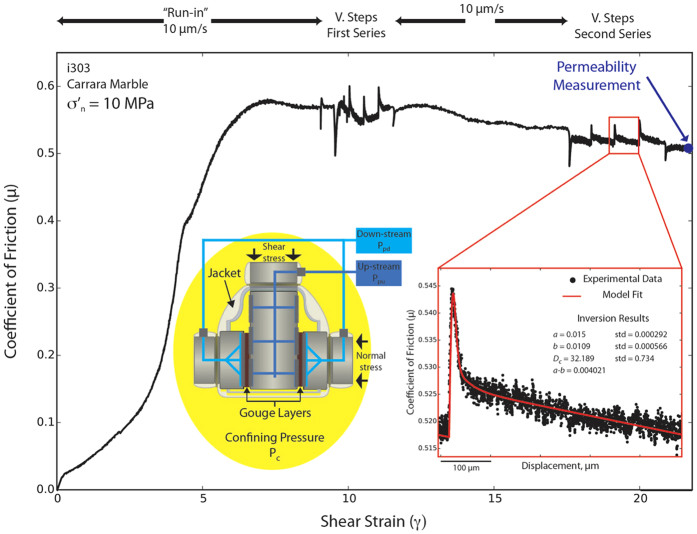
Friction experiments. Coefficient of friction vs. shear strain, γ, for one representative experiment on Carrara marble gouge. Velocity steps, for the characterization of the rate and state friction parameters, are conducted after about 7–10 γ (i.e 1 cm of shear displacement) and 17–21 γ (i.e. 2.5 cm of displacement). Permeability measurements are performed at the end of shearing. The inset in red shows the detail of one velocity step with the comparison between experimental data (black) and the result from the inversion model (red) used to obtain the (a-b) and *D*_*c*_ values. The yellow inset shows a schematic representation of the double direct shear configuration with forcing blocks equipped with fluid pressure conduits and the jacket to separate fluid pressurized gouge layers from the confining medium (details in the method).

We sheared granular layers of Carrara marble and limestone fault gouge, with a grain size <125 μm, in a double direct shear configuration within a pressure vessel to allow true-triaxial stress field (Fig. 1 inset and methods for additional details)^20^. Experiments on these two lithologies allow the comparison between the frictional behaviour of a standard material as Carrara marble generally used in rock deformation experiments, with a similar fault gouge collected along a natural fault. In laboratory experiments granular powders are usually used as analogue for fault gouge material formed by wear of two frictional interfaces. In order to explore frictional stability at different levels of fluid pressure (P_f_), confining pressure (P_c_) and applied normal stress (σ_n_) were maintained constant throughout a series of experiments and pore fluid pressure was increased from sub-hydrostatic (pore fluid factor λ = P_f_/σ_n_ = 0.15), supra-hydrostatic (λ = 0.5) to near-lithostatic (λ = 0.8)^4^ (Table S1). To evaluate frictional stability we performed two series of velocity stepping tests, with shear velocities from 0.1–100 μm/s, at different levels of shear strain (Fig. 1). In the following, we will present data for the velocity step sequences performed at higher strains since they are more representative of natural faults, where strain is generally high. At the end of each experiment we measured the resulting fault permeability (Fig. 1 and methods for additional details).

**Figure 2:**
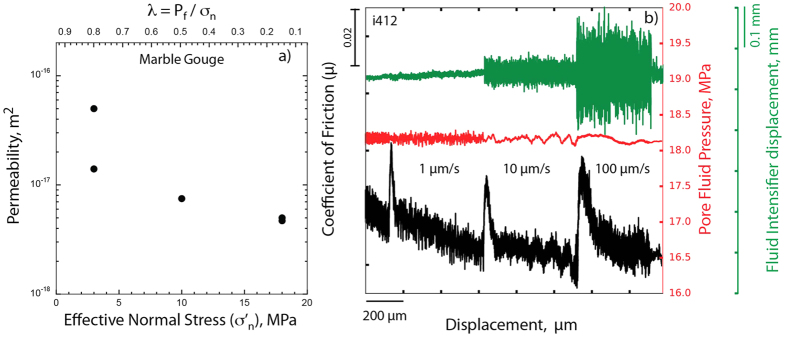
Fluid flow properties. **(a**) Permeability measurements on Carrara marble gouge at constant applied normal stress (σ_n_ = 21 MPa) and different values of pore fluids pressure, P_f_, resulting in different effective normal stresses, σ’_n_, and pore fluid factor λ = P_f_/σ_n_. (**b**) During velocity steps, the fluid pressure and the upstream intensifier displacement remain constant, indicating fully drained boundary conditions.

Our permeability measurements on Carrara marble at confining pressure of 19 MPa and under different level of fluid pressure, are in the range of 5 × 10^−17^ to 4.7 × 10^−18 ^m^2^ (Fig. 2a). Permeability increases with decreasing effective normal stress, as previously documented in numerous experiments on intact cylindrical samples^21^ and some experiments on powdered gouge material^18^. Our permeability measurements obtained after fabric development, i.e. after shearing the experimental fault for about 2.5 cm (Fig. 1), imply that during shearing the permeability is likely to be higher due to higher steady state porosity^22^. Permeability values >10^−17^ m^2^ greatly facilitates fluid movement through the fault^23^, suggesting that the experimental fault is in a fully drained condition. This is supported by the constant values of the pore fluid pressure monitored during the velocity steps (Fig. 2b and S1). Therefore, in our experiments we rule-out significant transient decreases or increases in fluid pressure resulting from dilation strengthening^9^ or compaction creep^24^ mechanisms respectively, that may influence friction measurements.

The stable sliding friction coefficient, μ_ss_, measured at the end of the run-in phase (Fig. 1) is nearly constant for all the tested boundary conditions and fault materials, and is in the range of 0.55 < μ < 0.60. This value is consistent with previous experimental results on carbonates^25^,^26^ and with strong crustal seismogenic faults^23^,^27^.

**Figure 3:**
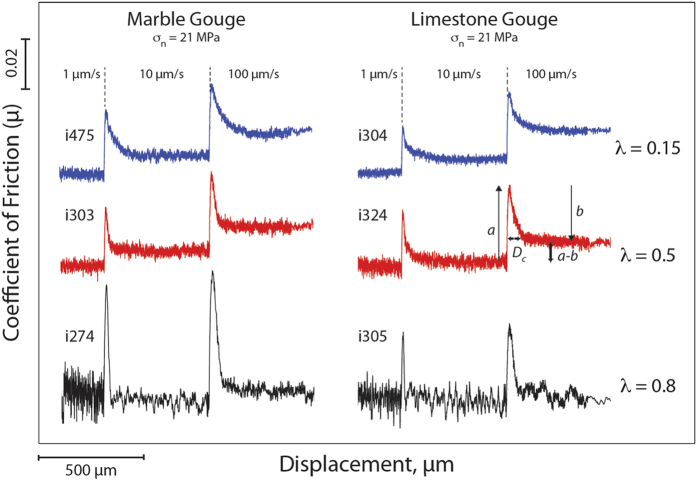
Fluid pressure and rate and state friction parameters. Raw data of experiments conducted on Carrara marble (left column) and limestone gouge (right column) at constant normal stress, σ_n_ = 21 MPa and different levels of fluid pressure: sub-hydrostatic, λ = 0.15, supra-hydrostatic λ = 0.5, near lithostatic, λ = 0.8. Note the transition from velocity strengthening (*a* > *b*) to velocity neutral (*a *≈ *b*) and the reduction of *D*_*c*_ with increasing fluid pressure.

**Figure 4:**
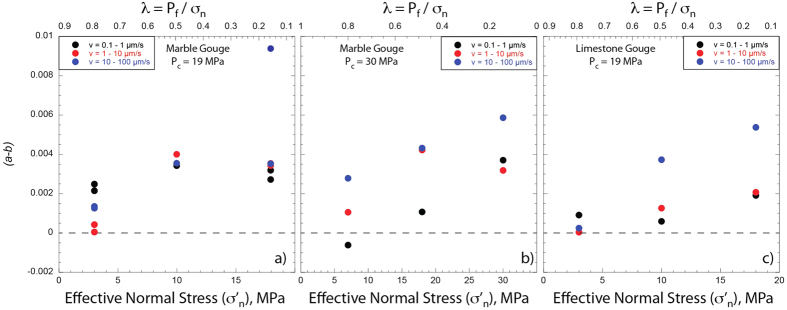
(*a-b*) vs. pore fluid pressure. Evolution of the (*a-b*) friction parameter as a function of the effective normal stress, σ’_n_, and pore fluid factor λ. Marble gouge at 19 MPa (**a**) and 30 MPa (**b**) of confining pressure, P_c_. Limestone gouge at 19 MPa (**c**) of confining pressure.

**Figure 5:**
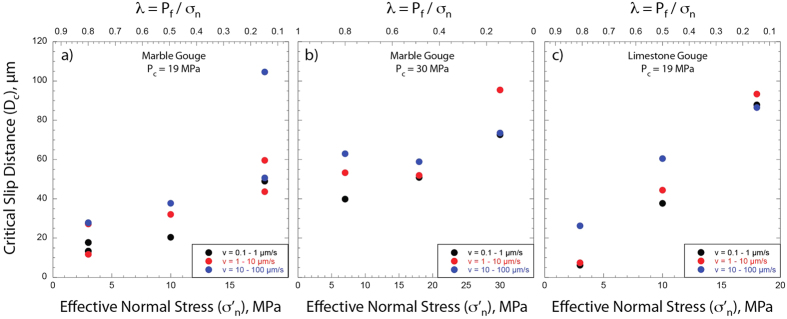
Critical slip distance, *D*_*c*_, vs. pore fluid pressure. Evolution of *D*_*c*_ as a function of the effective normal stress, σ’_n_, and pore fluid factor λ. Marble gouge at 19 MPa (**a**) and 30 MPa (**b**) of confining pressure, P_c_. Limestone gouge at 19 MPa (**c**) of confining pressure.

Figure 3 shows the comparison of raw data for velocity steps during 6 experiments conducted on both Carrara marble and limestone gouge at the same confining pressure and normal stress, but under different levels of pore fluid pressure, i.e. from sub-hydrostatic (blue curves), supra-hydrostatic (red curves) to near lithostatic (black curves) (Table S1). With increasing fluid pressure we observe an evolution from *a* > *b* (velocity strengthening) to *a* ≈ *b* (velocity neutral) and an important reduction in *D*_*c*_. This behaviour is observed across the entire dataset (Table S1). We modelled each velocity step using an iterative singular value decomposition technique to determine rate-and-state friction constitutive parameters (Fig. 1 inset and methods for details). The values of *(a − b)* evolve from velocity strengthening behaviour (*a − b* ≈ 0.005) at fluid pressure condition of sub-hydrostatic to a velocity neutral behaviour (*a − b* approaching 0), when the fault is at near lithostatic fluid pressure (Fig. 4). In general, this trend is observed at different confining pressures (Fig. 4a vs. 4b) and for different gouge material (Fig. 4a,b vs. 4c). Similarly, the constitutive parameter *D*_*c*_ consistently decreases as the pore fluid pressure is increased for all the gouge materials and boundary conditions tested^28^ (Fig. 5). We document an evolution from *D*_*c*_ ~ 90 μm, at sub-hydrostatic pore fluid pressure, to *D*_*c*_ ~ 10 μm, when pore pressure reaches near lithostatic conditions. The larger reduction in D_c_ is observed for limestone gouge (Fig. 5c). To discern if the reduction in *(a − b)* and *D*_*c*_ observed at near-lithostatic pore fluid pressure was caused only by the reduction in effective normal stress we performed additional tests at constant effective normal stress of 3 MPa and varying the pore fluid factor (Fig. S2 and Table S1). The comparison between experiments suggests that the reduction of *(a − b)* from slightly velocity strengthening to velocity neutral is facilitated by the increase of fluid pressure and associated with a reduction in layer dilation (Fig. S3). Whereas, the reduction in *D*_*c*_ observed at near-lithostatic pore fluid pressure (Fig. 5) is mainly controlled by the applied normal stress (Fig. S2). We also tested the experimental reproducibility of our data and found consistent values across different experiments performed at the same boundary conditions (Table S1). For instance, we report two experiments performed on marble gouge at confining pressure of 19 MPa and λ = 0.8, showing the same *(a − b)* and *D*_*c*_ values (Fig. 4a).

## Discussion

We have investigated the role of fluid pressure in frictional stability of carbonate-bearing faults for two main reasons. First, a great number of earthquakes nucleate or propagate through thick sequences of carbonates that dominate the upper-crustal sedimentary sequences. Examples include the 1995, Aigion earthquake M = 6.2 (ref. 29), the Umbria-Marche 1997–1998 sequence, M = 6.0 (refs 5 and 30), the 2009 L’Aquila sequence M = 6.1 (ref. 31), the 2012 Emilia sequence, M = 5.9 (ref. 32) and numerous earthquakes occurring in the Zagros Mountains 4.0 < M < 6.0 (ref. 33). In many of these seismic sequences fluid-pressure is thought to play a key-role in earthquake triggering^5^,^34^,^35^,^36^. Second, carbonate reservoirs contain half of the known conventional oil reserves. Within these reservoirs, ancient fault structures can be reactivated during wastewater fluid disposal, resulting in earthquakes. Examples of wastewater-induced seismicity that reactivate portions of carbonate-bearing faults have been documented in southern Italy^37^, Texas^38^ and Oklahoma^13^.

For carbonate-bearing fault gouges, our mechanical data show that the rate and state friction parameters do change with increasing fluid pressure (Figs 3, 4, 5). From sub hydrostatic to near lithostatic fluid pressure conditions the friction rate parameter *(a − b)* evolves from slightly velocity strengthening to velocity neutral. This occurs for both Carrara marble and limestone gouges and for different values of confining pressure (Fig. 4). The critical slip distance, *D*_*c*_, decreases with increasing fluid pressure from about 90 to 10 μm (Fig. 5). Since *D*_*c*_ is the distance to slide for contact surfaces to renew and it is related to the size of the contact junctions^7^,^39^,^40^,^41^, with decreasing the effective normal stress the contact area at the grain junctions is reduced^42^. The reduction in the contact area would promote a shorter *D*_*c*_ at lower effective normal stress. This implies that the applied normal stress rather then fluid pressure plays a key-role in the reduction of *D*_*c*_ as it is shown by our experimental data showing similar *D*_*c*_ obtained for the same effective normal stress (3 MPa) but at different fluid pressure levels (Fig. S2). However, it is worth noting that the increase of fluid pressure along a fault at seismogenic depth is the primary mechanism allowing the reduction of the applied normal stress that promotes shorter *D*_*c*_.

**Figure 6:**
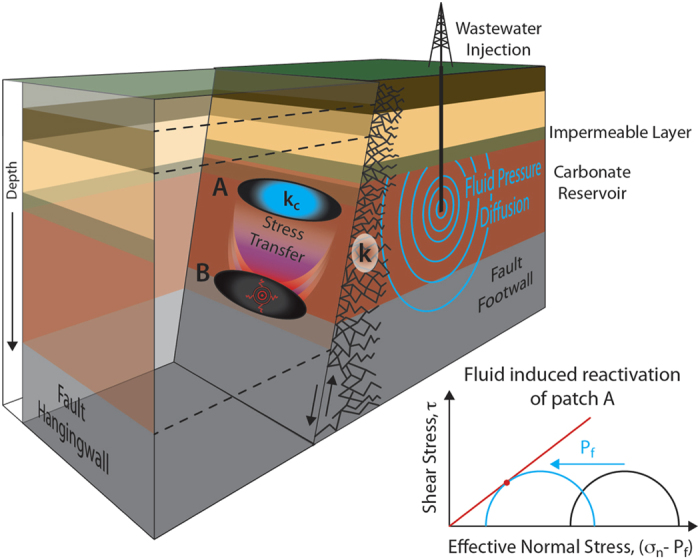
Schematic representation of fault reactivation due to fluid overpressure. Two end-members of fault slip behavior promoted by fluid assisted fault reactivation of patch A. Case 1) aseismic reactivation of patch A (slightly velocity strengthening) causes stress transfer and earthquake triggering on patch B (velocity weakening). Case 2) induced seismicity on patch A that due to fluid overpressure has a small *D*_*c*_ and a velocity neutral behavior.

Upon fluid induced fault reactivation^1^,^6^, for a fault-loading medium with a constant stiffness (k in equation 1) and for a fault patch possessing the rate and state friction parameters measured in our laboratory experiments, we propose the occurrence of two potential end-member fault slip behaviours (Fig. 6). In the first case, because the gouge is not velocity weakening, an increase in fluid pressure would promote fault creep, resulting in stress transfer and earthquake triggering on adjacent fault patches that could be more prone to develop frictional instabilities^43^. For carbonate-bearing faults, unstable fault slip might result from velocity weakening gouge at higher temperature^19^,^26^, sharp and localized slipping zones^44^ or strongly cemented fault portions^25^. This behaviour has been observed during an experiment along a well instrumented natural carbonate-bearing fault, where the increase of fluid pressure, induced by fluid injection, promoted aseismic slip along the fault, with induced microseismicity, as secondary effect, on adjacent regions^45^,^46^. In the second case, frictional instability can nucleate on a slightly velocity-strengthening/velocity-neutral fault gouge under appropriate boundary conditions. In the case of a single degree of freedom 1-D fault, obeying rate-and-state friction, Boatwright and Cocco (1996)^47^ have shown that even if the material is velocity strengthening: 1) an abrupt perturbation of the surrounding stress field, due to fluid pressure build up or poroelastic effects^48^, can drive a frictional instability, and 2) a reduction in *D*_*c*_, as documented in our experiments (Figs 3 and 5), further contributes to a transition from stable sliding to frictional instability. Once the fault has been reactivated by fluid overpressure, another factor that might promote frictional instabilities is fault slip weakening. With accumulated slip, the rate of frictional weakening, e.g.^12^, can overcome the slightly velocity strengthening/velocity neutral behaviour of calcite gouge hence promoting seismic slip. This second potential end-member fault slip behaviour is consistent with the observation that areas affected by deep wastewater injections are more susceptible to earthquake triggering from transient stresses due to high fluid pressure^49^.

Our data show that the increase of fluid pressure strongly influences the evolution of the rate-and-state friction parameters and consequently the fault rheological stiffness (k_c_ in eq. 1). Similarly, the stiffness of the loading medium (k in eq. 1) is likely influenced by fluid pressure build-ups during the circulation of crustal fluids. Other factors that influence fault slip stability during fluid pressure build-up include stress re-distribution^5^,^48^ and weakening effects during fault slip^12^. Therefore we suggest that it is restrictive to infer the fault slip behaviour only by differentiating between velocity strengthening or velocity weakening material^47^,^50^,^51^, in particular for materials, like those tested in this work, showing that with increasing fluid pressure the transition from velocity strengthening to velocity weakening become subtle. Finally, a better characterization of the frictional stability parameters (eq. 1) as a function of fluid pressure may prove useful for better defining the seismic potential of faults, implementing models of seismic hazard evaluation and better assessing the risk of human induced earthquakes.

## Method

**Figure 7:**
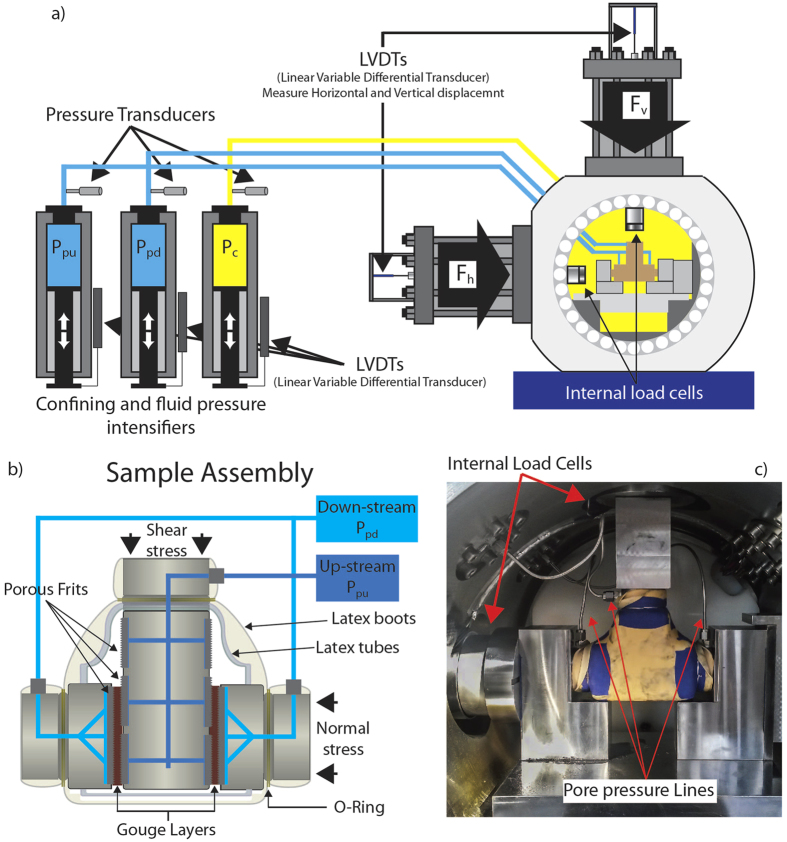
Experimental configuration. (**a**) BRAVA apparatus showing the double direct shear configuration within the pressure vessel and the intensifiers used to pressurize pore fluid (P_pu_, P_pd_) and confining pressure (P_c_). (**b**) Details of the sample assembly in the double direct shear configuration for vessel experiments. (**c**) Initial set-up showing the jacketed sample assembly with pore fluid pressure tubing and the internal load cells within the pressure vessel.

We performed double-direct shear experiments in a biaxial deformation apparatus (BRAVA, Brittle Rock deformAtion Versatile Apparatus at INGV HP-HT laboratory, Rome)^20^ equipped with a pressure vessel to allow a true-triaxial stress field (Fig. 7). A fast acting servo-hydraulic system was used to control applied stresses and/or displacements. The applied normal stress was maintained constant via a load-feedback servo control loop. Similarly, shear stress was applied via a controlled shear displacement rate imposed at the fault boundaries using servocontrol. Forces were measured using strain gauged load cells (manufactured by LEANE International model CCDG-0.1–100-SPEC), positioned inside the pressure vessel, with an amplified output of ±5 V and an accuracy of ±0.01 kN, which are calibrated regularly. The load cells are designed with central hollow in order to equilibrate the confining pressure when it is applied. This design allows us to measure vertical and horizontal load independently of the applied confining pressure and reach boundary conditions (i.e. near lithostatic) never reached in this configuration in previous laboratory experiments. Displacements were measured via Linear Variable Displacement Transformers (LVDT’s), with an accuracy of ±0.01 μm, referenced at the load frame and the upper side of the ram (Fig. 7a). Load point displacement measurements are corrected for the stiffness of the testing apparatus, with nominal values of 386.12 kN/mm for the vertical frame and 329.5 kN/mm for the horizontal frame. The pressure vessel is accessed via tubing that connect the inside of the chamber with three intensifiers to allow the application of an up- and down- stream pore fluid pressure to the fault zone and a confining pressure around it. Pore fluid and confining pressure are servo-controlled using fast-acting hydraulic servocontrollers. Confining pressure is applied using a hydrogenated, paraffinic white oil (XCELTHERM 600, Radco Industries), and maintained constant throughout the test using a load-feedback control mode. For pore fluid we used tap water, with a calcium rich chemical composition similar to the water circulating within carbonate bearing faults, and monitored pressure with diaphragm pressure transducers accurate to ±7 kPa.

The double direct shear configuration consists of a three forcing bocks assembly, with a central forcing block and two side stationary blocks that sandwich two layers of gouge material (Fig. 7b). Forcing blocks are equipped with high pressure fittings and internal conduits that provide fluid access to the gouge layers via sintered, stainless steel porous frits with permeability, *perm* ~ 10^−14 ^m^2^, which is high when compared with the permeability of the gouge layers (10^−17^ < *perm* < 10^−18 ^m^2^) (Fig. 2a). The frits are press fit into the forcing blocks and used to homogenously distribute fluids to the gouge layer boundaries. Frits were machined with grooves using an EDM technique to avoid damaging the pore structure; grooves are 0.8 mm in height with 1-mm spacing and oriented perpendicular to the shear direction to ensure that shear occurs within the gouge layers and not at the layer boundaries. The nominal frictional contact area is 5.54 cm × 5.55 cm, and we refer all measurements of stress, displacement, fluid volume and pressure changes to one layer. For these sample dimensions and loading configuration, normal stress on the gouge layers is determined by the summation of applied stress (σ_n_) and confining pressure (P_c_), with the effective normal stress acting on the gouge layers given by:


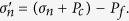


Gouge layers were prepared using levelling jigs to obtain a uniform initial layer thickness of 5 mm for all the experiments. In order to separate the gouge layer from the confining oil, building on the Penn State Rock and Sediment Mechanics Laboratory experience^18^,^52^,^53^, the sample assembly was sealed with a flexible latex jacket. To jacket the sample assembly (gouge layers + forcing blocks) we follow a three steps procedure: 1) a thick (~4 mm) flexible rubber sheet is secured around the blocks to provide support to the gouge layers for the successive steps; 2) two layers of latex rubber tubes were used to cover the frits exposed on the central forcing block to avoid any damage of the main jacket during the experiment; 3) two custom made latex boots, resembling the shape of the sample assembly, are used to cover the entire assembly (Fig. 7b). The sample assembly was then sealed with steel wires around the final latex boot, at the position of the O-rings positioned on the forcing blocks. At this stage high pressure fittings and tubing are connected to the forcing blocks, and the assembly placed within the pressure vessel (Fig. 7c).

Each experiment followed a common experimental procedure for reproducibility and comparison purposes. We started by applying the confining pressure in displacement feedback control until a pressure of 1 MPa was reached. At this stage the intensifier was switched to a load-mode feedback control and P_c_ was increased at steps of 1 MPa every 2 minutes, to give time to the layers to compact adequately, until the target value was reached. At this stage the horizontal piston was advanced in displacement feedback control until a horizontal force of about 0.4 MPa is reached, then switched in load-mode feedback control and the target normal stress was reached. The pore fluid pressure (P_f_) was then increased to 1 MPa from the up-stream intensifier (P_pu_), with the down-stream intensifier (P_pd_) left open to the atmosphere, until flow through the gouge layer was established. Once we ensured that gouge layers were fully saturated and all the residual air in the gouge was expelled, the down-stream intensifier was closed to the atmosphere, and left to equilibrate with the P_pu_. Pore fluid pressure was then increased with steps of 1 MPa every 5–10 minutes to the target value. The sample was left to equilibrate for about 30 minutes until it reached a steady layer thickness, which is indicative that the gouge layers reached the best packing configuration under the stress field applied before shear.

Layers were subject to shear loading by driving the central block of the double direct shear assembly at constant displacement rate. As shear stress first began to increase the sample jacket and rubber sheets that extend under the side forcing blocks flatten. We account for this elastic compaction via an elastic correction. Experiments were conducted using a computer-controlled displacement history (Fig. 1). Shearing began with an initial phase at 10 μm/s for ~10 mm (shear strain of 6–8), which served to condition the layers, localize shear and establish a steady state value of sliding friction. Then, we imposed two series of velocity step tests, from 0.1–1–10–100 μm/s, separated by 5 mm of shear at constant velocity of 10 μm/s, to investigate the evolution of the rate and state dependence of friction. At the end of each experiment we measured the resulting layer permeability, during a hold period, using a constant head method. During this test we impose a differential pressure between the up- and down- stream pore fluid intensifiers (usually 1 MPa) and measure the resulting flow rate across the gouge layers. We calculated permeability using Darcy’s law:


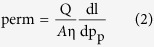


where *perm* is the sample permeability [m^2^], *Q* is the measured flow rate [m^3^s^−1^], A is the cross-sectional area [m^2^], η is the viscosity of water [MPa s], ∆P_p_ is the imposed differential pore pressure [MPa], and *d*l is the sample length. We assume η = 1.002 × 10^−9 ^MPa s^−1^, and define *d*l from the horizontal displacement record and *Q* as the average value of the flow rates measured at the up-stream (P_pu_) and down-stream (P_pd_) pumps. To ensure steady state flow conditions, we waited until the flow rate difference, between Q_u_ and Q_d_, was less than 5%.

To investigate friction constitutive behaviour and fault slip stability we modelled experimental data from velocity step tests using the rate-and-state friction constitutive equations^7^,^8^:


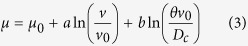



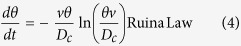


where μ_0_ represents the reference coefficient of friction at velocity *v*_*0*_, *v* is the frictional slip rate, *a* and *b* are empirical constants, *D*_*c*_ is the critical slip distance and θ is the state variable, representing the contacts average life time. To model details of frictional evolution, eq. 3 must be coupled with a description of the state evolution (eq. 4)^10^. For our modelling we choose the slip evolution law proposed by Ruina (1984)^8^. In order to take in account for the finite stiffness of our experimental apparatus and its elastic interaction with the gouge layers, we couple eqs 3 and 4 with the time derivative of a simple spring equation:


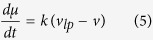


where *v*_*lp*_ is the load point velocity and *k* is the stiffness (given in units of μ^−1^) measured from the loading slope of velocity steps^54^,^55^,^56^. Because *k* can slightly vary as a function of confining pressure, we determined a single value of *k*, usually in the range 0.005 < k < 0.008 μ^−1^, for each experiment and used it for all the inversions concerning those data. To obtain rate-and-state parameters *a, b* and *D*_*c*_, we solve eqs 3 and 4 using a fifth-order Runge-Kutta numerical integration technique with adaptive step-size, with eq. 5 as a constraint. The best-fit values of the constitutive parameters are determined using an iterative, least-square method. For a typical model fit the unweighted chi square error is usually ≤0.0001, and the variance is ≤5 × 10^−7^. The estimated error is calculated from the covariance matrix and expressed as one standard deviation, which is usually ≤0.0002. These errors are usually smaller than the uncertainties associated with experimental reproducibility, which we tested by repeating experiments under identical boundary condition (Table S1).

## Additional Information

**How to cite this article**: Scuderi, M. M. and Collettini, C. The role of fluid pressure in induced vs. triggered seismicity: insights from rock deformation experiments on carbonates. *Sci. Rep.* **6**, 24852; doi: 10.1038/srep24852 (2016).

## Supplementary Material

Supplementary Information
