# Supplementary material for: The role of fluid pressure in induced vs. triggered seismicity: insights from rock deformation experiments on carbonates
Source: Sci Rep. 2016 Apr 26;6:24852. doi: 10.1038/srep24852 (PMC4845004; doi:10.1038/srep24852)
Supplement: Supplementary Information [file srep24852-s1.pdf]

The role of fluid pressure in induced vs. triggered seismicity: insights from rock deformation experiments on carbonates

Marco M. Scuderi[1,2*] and Cristiano Collettini[1,2]

[1]*Dipartimento di Scienze della Terra, Sapienza Università di Roma, Piaz. Aldo Moro 5, 00187 Rome, Italy*

[2]*Istituto Nazionale di Geofisica e Vulcanologia, INGV, via di Vigna Murata 605, 00143 Rome, Italy*

** Corresponding author: marco.scuderi@uniroma1.it*

Supplementary Material

Contains Extended Data Figure 1, 2, 3

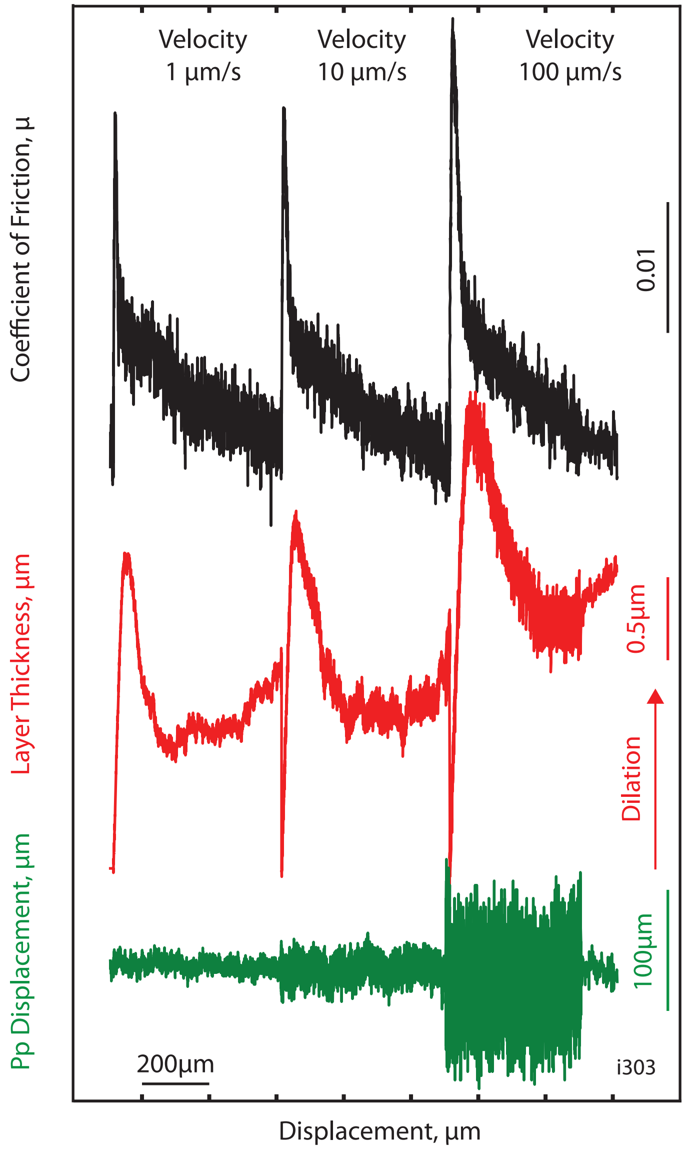

Extended Data Figure 1. Experimental curves showing the evolution of friction (black) layer thickness (red) and up-stream pore fluid pressure displacement (green) during a series of velocity steps from 0.1 to 100 µm/s.

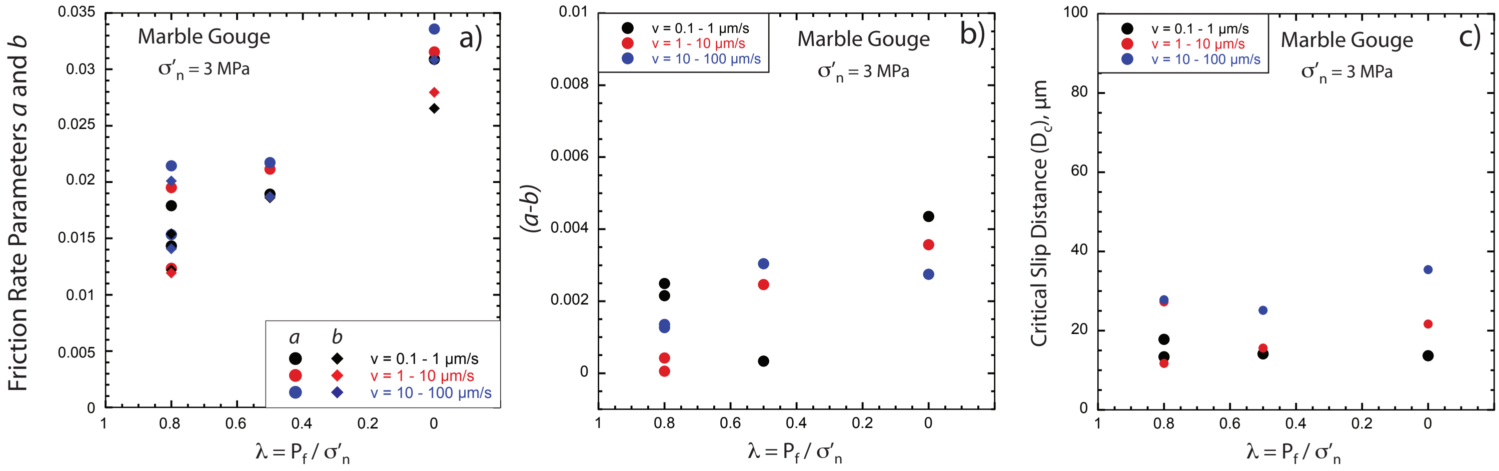

Extended Data Figure 2. (a) Friction rate parameters *a* and *b*; (b) *(a-b)* and (c) Dc obtained for the same effective normal stress (3 MPa) but at different fluid pressure levels as expressed by the pore fluid factor, λ.

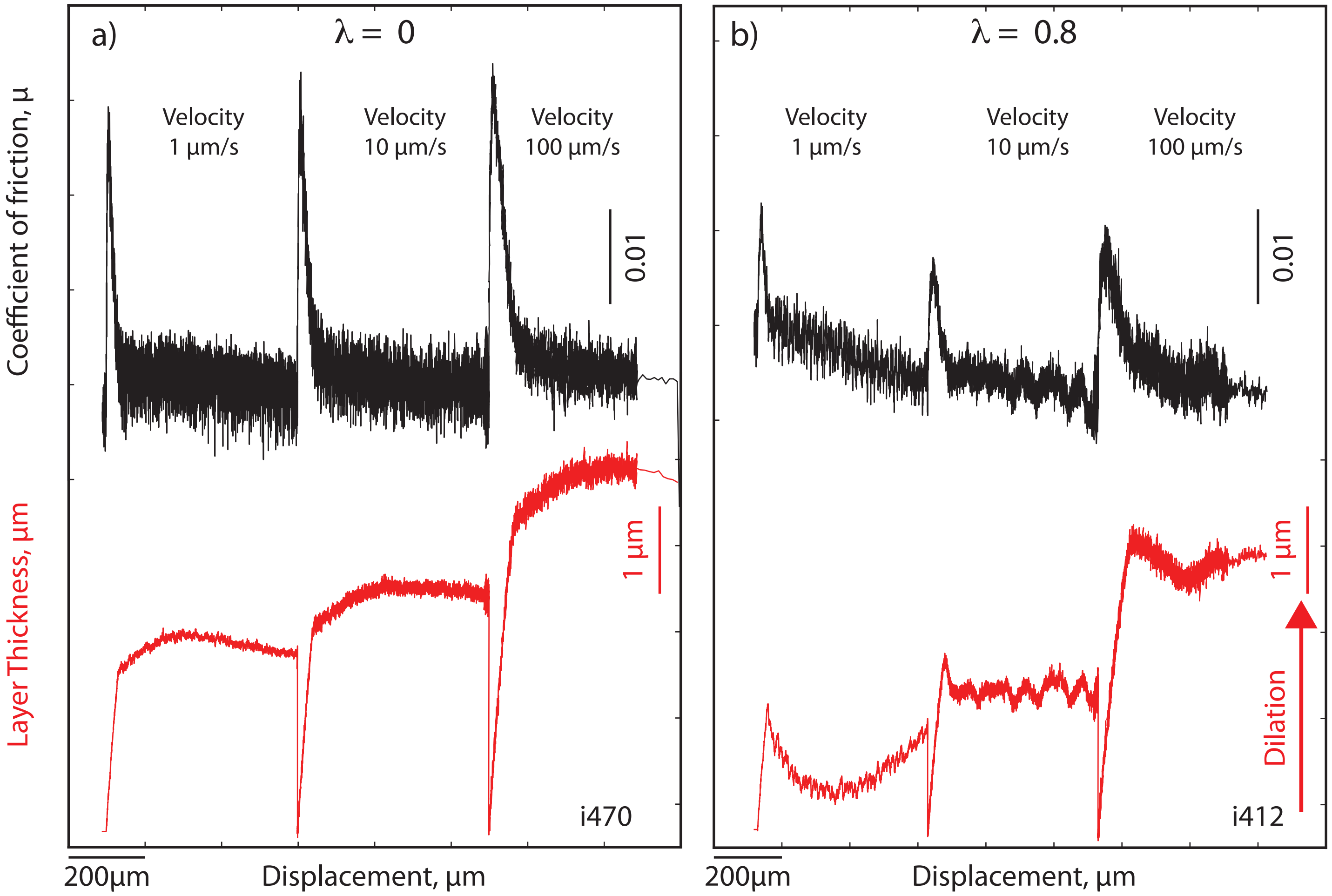

Extended Data Figure 3. Evolution of friction (black) and layer thickness (red) during velocity steps for experiments conducted at (a) $\lambda = 0$ and (b) $\lambda = 0.8$ and the same effective normal stress of 3 MPa.

Exp. Num.	Gouge Material	σ_n (MPa)	P_c (MPa)	P_f (MPa)	σ'_n (MPa)	λ (P_f / σ_n)	Velocity Steps (μm/s)	*(a-b)*	*Dc*
i274	Carrara Marble	2	19	18	3	0.85	10 – 1<br>1 – 10<br>10 – 100	0.002489<br>5.3e-5<br>0.001355	17.788<br>11.738<br>27.846
i412	Carrara Marble	2	19	18	3	0.85	0.1 – 1<br>1 – 10<br>10 – 100	0.002155<br>0.000422<br>0.001268	13.399<br>27.318<br>30.174
i413	Carrara Marble	2	4	2.5	3	0.5	0.1 – 1<br>1 – 10<br>10 – 100	0.000334<br>0.002464<br>0.003044	14.178<br>15.655<br>25.168
i470	Carrara Marble	3	0	0<br>Saturated	3	0	0.1 – 1<br>1 – 10<br>10 – 100	0.004352<br>0.003571<br>0.002752	13.682<br>21.669<br>36.079
i303	Carrara Marble	2	19	11	10	0.5	0.1 – 1<br>1 – 10<br>10 – 100	0.003505<br>0.004021<br>0.003561	20.469<br>32.189<br>37.886
i475	Carrara Marble	2	19	3	18	0.14	0.1 – 1<br>1 – 10<br>10 – 100	0.002727<br>0.003553<br>0.003542	49.180<br>43.817<br>104.94
i273	Carrara Marble	2	19	3	18	0.14	10 – 1<br>1 – 10<br>10 – 100	0.003200<br>0.003442<br>0.009416	50.345<br>59.750<br>50.823
i355	Carrara Marble	5	30	28	7	0.8	0.1 – 1<br>1 – 10<br>10 – 100	-0.00061<br>0.001056<br>0.002788	39.841<br>53.309<br>63.014
i359	Carrara Marble	5	30	18	17	0.5	0.1 – 1<br>1 – 10<br>10 – 100	0.001075<br>0.004223<br>0.004320	50.885<br>51.968<br>58.870
i358	Carrara Marble	5	30	5	30	0.14	0.1 – 1<br>1 – 10<br>10 – 100	0.003707<br>0.003186<br>0.005867	72.658<br>95.465<br>73.544
i305	Limestone Gouge	2	19	18	3	0.85	0.1 – 1<br>1 – 10<br>10 – 100	0.000914<br>3.80e-05<br>0.000253	6.2066<br>6.7239<br>26.281
i324	Limestone Gouge	2	19	11	10	0.5	0.1 – 1<br>1 – 10<br>10 – 100	0.000591<br>0.001266<br>0.003726	37.667<br>44.377<br>60.479
i304	Limestone Gouge	2	19	3	18	0.14	0.1 – 1<br>1 – 10<br>10 – 100	0.001912<br>0.002072<br>0.005374	87.908<br>93.415<br>86.515

Extended Data Table 1. Summary of experiments and boundary conditions
